# PD 0332991, a selective cyclin D kinase 4/6 inhibitor, preferentially inhibits proliferation of luminal estrogen receptor-positive human breast cancer cell lines *in vitro*

**DOI:** 10.1186/bcr2419

**Published:** 2009-10-29

**Authors:** Richard S Finn, Judy Dering, Dylan Conklin, Ondrej Kalous, David J Cohen, Amrita J Desai, Charles Ginther, Mohammad Atefi, Isan Chen, Camilla Fowst, Gerret Los, Dennis J Slamon

**Affiliations:** 1 Department of Medicine, Division of Hematology/Oncology, Geffen School of Medicine at UCLA, 10833 Le Conte Ave, 11-934 Factor Bldg, Los Angeles, CA 90095, USA; 2 Pfizer Global Research and Development, Pfizer Inc., 10724 Science Center Drive, San Diego, CA 92121, USA; 3 Pfizer Oncology BU, Clinical Development, Pfizer Inc., Via Lorenteggio 257, Milan 20152, Italy

## Abstract

**Introduction:**

Alterations in cell cycle regulators have been implicated in human malignancies including breast cancer. PD 0332991 is an orally active, highly selective inhibitor of the cyclin D kinases (CDK)4 and CDK6 with ability to block retinoblastoma (Rb) phosphorylation in the low nanomolar range. To identify predictors of response, we determined the *in vitro *sensitivity to PD 0332991 across a panel of molecularly characterized human breast cancer cell lines.

**Methods:**

Forty-seven human breast cancer and immortalized cell lines representing the known molecular subgroups of breast cancer were treated with PD 0332991 to determine IC_50 _values. These data were analyzed against baseline gene expression data to identify genes associated with PD 0332991 response.

**Results:**

Cell lines representing luminal estrogen receptor-positive (ER+) subtype (including those that are HER2 amplified) were most sensitive to growth inhibition by PD 0332991 while nonluminal/basal subtypes were most resistant. Analysis of variance identified 450 differentially expressed genes between sensitive and resistant cells. pRb and cyclin D_1 _were elevated and CDKN2A (p16) was decreased in the most sensitive lines. Cell cycle analysis showed G_0_/G_1 _arrest in sensitive cell lines and Western blot analysis demonstrated that Rb phosphorylation is blocked in sensitive lines but not resistant lines. PD 0332991 was synergistic with tamoxifen and trastuzumab in ER+ and HER2-amplified cell lines, respectively. PD 0332991 enhanced sensitivity to tamoxifen in cell lines with conditioned resistance to ER blockade.

**Conclusions:**

These studies suggest a role for CDK4/6 inhibition in some breast cancers and identify criteria for patient selection in clinical studies of PD 0332991.

## Introduction

Breast cancer is a worldwide health concern with approximately 1,000,000 million new cases each year [1]. Significant advances have been made in our understanding of this malignancy and several molecular subtypes of breast cancer have been characterized [2-4]. This molecular understanding has paved the way for the development of new agents that target pathogenic molecular alterations that drive tumor cell growth while sparing patients many of the traditional toxicities associated with chemotherapy. Ubiquitous to all cancer types is abnormal proliferation with dysregulation of normal cell cycle control [5]. For this reason, inhibitors of key cell cycle regulators are attractive targets for novel cancer therapeutics [6]. Successful clinical development of this class of agents, however, will require some understanding of which subgroup of patients will be more likely to benefit from these targeted interventions.

Under normal control, the cell cycle functions as a tightly regulated and predictable process consisting of several distinct phases: G_0 _(quiescence) followed by G_1 _(pre-DNA synthesis), S (DNA synthesis), G_2 _(pre-division), and M (cell division). The careful regulation of this system is of fundamental importance, and dysregulation can result in several disease processes including cancer. The progression from G_1 _to S is a key checkpoint in protecting the cell from abnormal replication. Key to passage through this restriction point is the interaction between the cyclin-dependent kinases (CDKs) and cyclin proteins. CDKs are a subgroup of serine/threonine kinases that play a key role in regulating cell cycle progression by associating with cyclins. Hyperphosphorylation of the retinoblastoma (Rb) gene product pRb is mediated in early G_1 _by CDK4 and CDK6 interacting with cyclin D_1_. This results in pRB inactivation and release of transcription factors that allow progression to the S phase. Negative regulators of CDK4/6-cyclin activity include the INK4 family (p16, p15, p18, p19) [7].

Several studies have identified alterations of cell cycle regulators in human breast cancer (reviewed in [8,9]) and provide a rationale for a potential therapeutic role for CDK4/6 inhibition in this tumor type. Amplification of the cyclin D_1 _gene has been identified in approximately 15 to 20% of human breast cancers [10,11] while overexpression of the protein has been demonstrated in a higher percentage [12,13]. The prognostic significance of cyclin D_1 _overexpression is not clear; some studies suggest it is a dominant oncogene associated with poor clinical outcomes [11,14-16], while other studies suggest it is associated with a more indolent, estrogen receptor (ER)-positive phenotype [17,18]. In addition, studies have associated cyclin D amplification with resistance to tamoxifen [19,20]. While the interaction between CDK4/6 and cyclin D_1 _suggests their interdependence, cyclin D_1 _has been found to function independently of CDK4/6 in supporting proliferation by directly activating ER [21,22]. Finally, loss of function of pRb has been described in 20 to 35% of breast cancers (reviewed in [23]).

The majority of CDK targeted agents to date have not focused on CDK4/6 targeting but rather on CDK1/2 targeting. Consequently the most advanced agents in development are aimed at these targets [24,25]. Further, limited data exist regarding the preclinical activity of CDK4/6 inhibitors in breast cancer. PD 0332991 is an orally active potent and highly selective inhibitor of CDK4 and CDK6 kinases, which in low nanomolar concentrations blocks pRb phosphorylation - subsequently inducing G_1 _arrest in sensitive cell lines [26-29]. Preclinical studies have demonstrated that PD 0332991 induces G_1 _arrest in primary bone marrow cells *ex vivo *and prevents tumor growth in disseminated human myeloma xenografts [30].

Based on the above biology, we hypothesized that there might be a molecular subgroup of human breast cancers that would be dependent on CDK4/6 function and would be likely to respond to this agent. Previous studies have demonstrated that *in vitro *large-panel analyses of molecularly characterized breast cancer cell lines can offer insight into the molecular heterogeneity of the clinical disease [31,32]. To identify potential biomarkers of response to PD 0332991 and to assist in patient selection and clinical development, we therefore evaluated the effects of PD 0332991 in a panel of 47 human breast cancer and immortalized breast cell lines growing *in vitro*.

## Materials and methods

### Cell lines, cell culture, and reagents

The cell lines used in the analysis include MDA-MB-415, MDA-MB-134, HCC-1500, ZR-75-30, HCC-202, HCC-1419, HCC-38, HCC-70, HCC-1187, HCC-1806, HCC-1937, HCC-1954, MDA-MB-436, HCC-1569, Hs578t, HCC-1143, MDA-MB-175, BT-474, SK-BR-3, MDA-MB-361, UACC-893, UACC-812, UACC-732, T-47D, MDA-MB-453, MDA-MB-468, CAMA-1, MDA-MB-157, MCF-7, MDA-MB-435, ZR-75-1, BT-20, MDA-MB-231, BT-549, DU4475, HCC-1395, HCC-2218, 184A1, 184B5 and MCF-10A, and were obtained from American Type Culture Collection (Rockville, MD, USA). The cell lines EFM-192A, KPL-1, EFM-19, COLO-824 and CAL-51 were obtained from the German Tissue Repository DSMZ (Braunschweig, Germany), and the cell lines SUM-190 and SUM-225 were obtained from the University of Michigan (Ann Arbor, MI, USA).

MDA-MB-134, MDA-MB-415, MDA-MB-436, MDA-MB-175, UACC-893, UACC-812, and MDA-MB-157 cells were cultured in L15 medium supplemented with 10% heat-inactivated FBS, 2 mmol/l glutamine and 1% penicillin G-streptomycin-fungizone solution (PSF) (Irvine Scientific, Santa Ana, CA, USA). CAL-51, KPL-1, and Hs578t cells were grown in DMEM (Cellgro, Manassas, VA, USA) supplemented with 10% heat-inactivated FBS and PSF, as above. SUM-190 and SUM-225 cells were cultured in HAM's F12 supplemented with 5% heat-inactivated FBS, PSF, 5 mg/ml insulin and 1 mg/ml hydrocortisone. 184A1, 184B5, and MCF 10A cells were grown in a 50/50 mix of mammary epithelial basal medium (MCDB 170) (US Biological, Swampscott, MA, USA) supplemented with 1.5 ml/l bovine pituitary extract (Invitrogen, Carlsbad, CA, USA), 20 μl/l epidermal growth factor (Invitrogen), 10 ml insulin (Sigma, Saint Louis, MO, USA), 1 ng/ml cholera toxin (Calbiochem, San Diego, CA, USA), 0.5 mg/l hydrocortisone (Sigma), and RPMI 1640 supplemented with 10% heat-inactivated FBS, 2 mmol/l glutamine, and 1% PSF. The remaining cell lines were cultured in RPMI 1640 (Cellgro) supplemented with 10% heat-inactivated FBS, 2 mmol/l glutamine, and 1% PSF. A tamoxifen-resistant MCF7 cell line was developed after serial passage in RPMI 1640 without phenol red (Invitrogen) supplemented with 10% charcoal-stripped FBS (Fisher Scientific, Pittsburgh, PA, USA) and 2 mmol/l glutamine, and PSF.

### Transcript microarray analyses

Briefly, cells were grown to log phase and then RNA was extracted using the RNeasy Kit (Qiagen, Valencia, CA, USA). The purified RNA was eluted in 30 to 60 μl diethylpyrocarbonate (DEPC) water and the quantity of RNA measured by spectral analysis using the Nanodrop Spectrophotometer (NanoDrop Products, Wilmington, DE, USA). RNA quality was determined by separation of the RNA via capillary electrophoresis using the Agilent 2000 Bioanalyzer (Agilent Technologies, Santa Clara, CA, USA). Microarray hybridizations of 51 breast cell lines were performed using the Agilent Human 1A V1 array.

Characterization of individual breast cancer cell line transcripts was performed by comparison with a breast cell line mixed reference pool of RNA and was conducted on a single slide in which the cell line mixture RNA was labeled with cyanine-3 and RNA from the individual cell line was labeled with cyanine-5. The mixed reference cRNA pool consisted of equal amounts of cRNA from nine breast cancer cell lines and one immortalized breast cell line selected to be representative of the full spectrum of breast cancer subtypes based on their expression of specific molecular markers - for example, ESR1, HER2, epidermal growth factor receptor, cytokeratins, and so forth - as well as growth characteristics. The reference cRNA pool includes RNA from 184B5, MDA-MB-468, MDA-MB-157, MDA-MB-231, MDA-MB-175, CAMA-1, MCF-7, MDA-MB-361, SK-BR-3, and DU4475 cell lines.

Microarray slides were read using an Agilent Scanner, and Agilent Feature Extraction software version 7.5 was used to calculate gene expression values. The feature extracted files were imported into the Rosetta Resolver^® ^system version 7.1 for gene expression data analysis (Rosetta Biosoftware, Seattle, WA, USA). The intensity ratios between the cell line sample and mixed reference calculated for each sequence were computed according to the Agilent error model. A particular sequence was considered differentially expressed if the calculated p-value of change was *P *≤ 0.01. These data are available with accession number [GEO:GSE18496].

### Proliferation assays

Cells were seeded in duplicate at 5,000 to 10,000 cells per well in 24-well plates. The day after plating, PD 0332991 was added at 1 μM and twofold dilutions over six concentrations were performed to generate a dose-response curve. Control wells without drug were also seeded. Cells were counted on day 1 when the drug was added as well as after 6 days when the experiment ended. After trypsinization, cells were placed in Isotone solution and counted immediately using a Coulter Z2 particle counter (Beckman Coulter Inc., Fullerton, CA, USA). Suspension lines were counted using a Coulter Vi-Cell counter (Beckman Coulter Inc.).

Growth inhibition was calculated as a function of the number of generations inhibited in the presence of PD 0332991 versus the number of generations over the same time course in the absence of PD 0332991. In addition, lethality was defined as any decrease in cell number in treated wells versus the baseline number of cells pre-treatment at day 1 of exposure.

For tamoxifen studies with the MCF7 tamoxifen-resistant cell line, proliferation studies were performed as above except cells were plated without FBS and were supplemented with 0.5 nM β-estradiol (Sigma). Proliferation assays were then performed as above.

### Multiple drug effects analysis

Similar to above, the ER-positive cell lines MCF-7, T47-D, and EFM-19 were plated and treated with PD 0332991 alone, with 4-hydroxytamoxifen (Sigma) alone, or with the combination, in duplicate, over six twofold dilutions at a fixed molar ratio. For combination studies with trastuzumab, BT-474, EFM-192A, and MDA-361 lines were plated as above and treated with PD 0332991 alone, with trastuzumab (Genentech, South San Francisco, CA, USA) alone, or with the combination, in duplicate, over six twofold dilutions at a fixed molar ratio.

For each assay, the log of the fraction growth inhibition was plotted against the log of drug concentration, and the linear regression curve fit correlation coefficient (*r *value) was calculated. Multiple drug effect analysis was performed using computer software as previously described [33].

Combination index (CI) values were derived from parameters of the median effects plots, and statistical tests were applied (unpaired, two-tail Student *t *test) to determine whether the mean CI values at multiple concentrations were significantly different from CI = 1. In this analysis, synergy is defined as CI values significantly lower than 1.0, antagonism as CI values significantly higher than 1.0, and additivity as CI values equal to 1.0. All CI values were calculated using the conservative assumption of mutually nonexclusive drug interactions. All experiments were carried out at least twice. Combination studies were performed as above with the MCF7 tamoxifen-resistant clones with addition of estrogen back to the media at the time of the experiment (as described in cell culture above).

### Western blot analysis

Cells in log-phase growth were treated with 100 nM PD 0332991 and were harvested at various timepoints by washing in PBS and lysis at 4°C in RIPA lysis buffer. Insoluble material was cleared by centrifugation at 10,000 × *g *for 10 minutes and protein was quantitated using bicinchoninic (BCA) (Pierce Biochemicals, Rockford, IL, USA). Protein content was resolved by SDS-PAGE electrophoresis, and was transferred to nitrocellulose membranes (Invitrogen). Total pRb expression was detected using a rabbit polyclonal antibody to pRb (Abcam, Cambridge, MA, USA). Rb phosphorylation was detected using rabbit polyclonal antibody to phospho-serine 780 (Cell-signaling, Danvers, MA, USA). Blots were washed and incubated with a goat-anti-rabbit IgG horseradish peroxidase conjugate (Upstate, Bellerica, MA, USA), developed using ECL Plus chemifluorescent reagent (Amersham Biosciences, Pistcataway, NJ), and imaged using chemifluorescence.

### Cell cycle analysis and apoptosis studies

Effects of PD 0332991 on the cell cycle were assessed using Nim-DAPI staining (NPE Systems, Pembroke Pines, FL, USA). Cells were plated evenly in control and experimental wells, were allowed to grow to log phase and were then treated with 100 nM PD 0332991 for the defined times. To perform cell cycle analysis, cells were washed with PBS; then trypsin was applied to release cells, which were then centrifuged at 10,000 × g for 5 minutes. Supernatant was aspirated and cells were then resuspended in 100 μl Nim-DAPI (NPE Systems) and gently vortexed.

Cells were analyzed with UV using a Cell Lab Quanta SC flow cytometer (Beckman-Coulter Inc.). Apoptosis assays were performed using an Annexin V-FITC apoptosis detection kit (MBL, Woburn, MA, USA) and flow cytometry. Cells were plated and treated as for cell cycle studies and were exposed to 100 nM PD 0332991 for 5 days. After incubation, cells were processed as directed in the kit and were analyzed using a FITC signal detector and propidium iodide detector using a Cell Lab Quanta SC flow cytometer.

### Statistical methods

Growth response to PD 0332991 and molecular subtype classification data were entered into the Statistica data analysis system version 8.0 (StatSoft Inc., Tulsa, OK, USA). The Pearson chi-square test was used to assess the relationship between response and subtype.

Breast cell lines were profiled on the Agilent Human 1A V1 platform that contains 17,086 probes including known genes and ESTs. The Resolver system analysis of variance (ANOVA) and hierarchical cluster analysis of the breast cell line expression profiles were used to compare the most sensitive cell lines (n = 21, IC_50 _< 150 nM) and the most resistant cell lines (n = 12, IC_50 _> 1,000 nM). All ANOVAs were performed using the Benjamini-Hochberg False Discovery Rate (FDR) multiple test correction and a statistical cutoff value for sequences of a twofold change in at least three experiments. The criteria used to determine differentially expressed genes were *P *< 0.05 with a difference between average expression for each group of at least |0.2|. Sequence sets were compared using the Venn Diagram tool in the Resolver system. The two-dimensional cluster analysis was performed using an agglomerative hierarchical clustering algorithm based on the cosine correlation similarity metric.

## Results

### PD 0332991 inhibits growth of luminal ER-positive as well as HER2-amplified breast cancer cell lines

A total of 44 human breast cancer cell lines and three immortalized breast epithelial lines were categorized as representing luminal or basal breast cancer subtypes based on the relative gene expression of cytokeratin 8/cytokeratin 18 and cytokeratin 5/cytokeratin 17, respectively [31,32].

**Figure 1:**
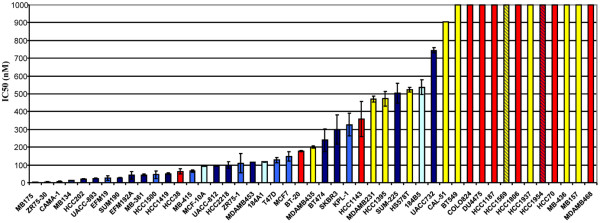
Inhibitory concentration and cell type. Bar graph of IC_50 _values (nM) and cell type. Cell lines are color coded by subtype: light blue, luminal; dark blue bars or stripes, HER2 amplified; yellow, nonluminal/undergone an epithelial-to-mesenchymal transition; red, nonluminal; turquoise, immortalized.

Several cell lines were further classified as representing breast cancers that had undergone an epithelial-to-mesenchymal transition based on their expression of vimentin and the loss of cytokeratin expression. Some cell lines that demonstrated mixed cytokeratin expression were classified as primarily basal due to the lack of expression of several additional well-characterized luminal markers, including ER, E-cadherin (CDH1), and GATA3. The calculated IC_50 _for each cell line and its molecular classification as well as the ER status and HER2 amplification status was determined (Table 1 and Figure 1).

**Table 1:** Cell lines arranged from most sensitive (low IC_50_) to least sensitive (high IC_50_)

Cell line	**IC**_50_**value (nM)**	**IC**_50_**standard error**	Breast cancer subtype	ER status	HER2 status
MDA-MB-175	4	0.4	Luminal	Positive	Normal
ZR-75-30	5	0.5	Luminal	Positive	Amplified
CAMA-1	8	0.4	Luminal	Positive	Normal
MDA-MB-134	13	1.3	Luminal	Positive	Normal
HCC-202	21	2.3	Luminal	Positive	Amplified
UACC-893	24	1.6	Luminal	Positive	Amplified
EFM-19	27	12.3	Luminal	Positive	Normal
SUM-190	28	1.3	Luminal	Positive	Amplified
EFM-192A	42	21.2	Luminal	Positive	Amplified
MDA-MB-361	44	4.1	Luminal	Positive	Amplified
HCC-1500	45	22.6	Luminal	Positive	Normal
HCC-1419	51	3.7	Luminal	Positive	Amplified
HCC-38	64	14.8	Basal	Negative	Normal
MDA-MB-415	64	6.6	Luminal	Positive	Normal
MCF-10A	92	0.1	n/a	Negative	Immortalized
UACC-812	96	4.6	Luminal	Positive	Amplified
HCC-2218	100	17.0	Luminal	Positive	Amplified
ZR-75-1	110	54.1	Luminal	Positive	Normal
MDA-MB-453	115	1.4	Luminal	Negative	Amplified
184A1	118	2.0	n/a	Negative	Immortalized
T47-D	127	15.0	Luminal	Positive	Normal
MCF-7	148	25.7	Luminal	Positive	Normal
BT-20	177	3.1	Basal	Negative	Normal
MDA-MB-435	201	7.5	Post-EMT	Negative	Normal
BT-474	240	64.5	Luminal	Positive	Amplified
SK-BR-3	300	83.0	Luminal	Negative	Amplified
KPL-1	327	64.3	Luminal	Positive	Normal
HCC-1143	359	99.7	Basal	Negative	Normal
MDA-MB-231	432	16.1	Post-EMT	Negative	Normal
HCC-1395	472	39.8	Post-EMT	Negative	Normal
SUM-225	503	55.7	Luminal	Negative	Amplified
Hs578t	524	12.3	Post-EMT	Negative	Normal
184B5	538	41.1	n/a	Negative	Immortalized
UACC-732	744	14.9	Luminal	Positive	Amplified
CAL-51	905	0.0	Post-EMT	Negative	Normal
BT-549	1,000	n/a	Post-EMT	Negative	Normal
COLO-824	1,000	n/a	Basal	Negative	Normal
DU4475	1,000	n/a	Basal	Negative	Normal
HCC-1187	1,000	n/a	Basal	Negative	Normal
HCC-1569	1,000	n/a	Post-EMT	Negative	Amplified
HCC-1806	1,000	n/a	Basal	Negative	Normal
HCC-1937	1,000	n/a	Post-EMT	Negative	Normal
HCC-1954	1,000	n/a	Basal	Negative	Amplified
HCC-70	1,000	n/a	Basal	Negative	Normal
MDA-MB-436	1,000	n/a	Post-EMT	Negative	Normal
MDA-MB-157	1,000	n/a	Post-EMT	Negative	Normal
MDA-MB-468	1,000	n/a	Basal	Negative	Normal

Included are molecular subtype and estrogen receptor (ER) and HER2 status. Cell lines representing the luminal ER-positive subtype were most sensitive to the growth inhibition effects of PD 0332991. n/a, not applicable; Post-EMT, cell lines classified as representing breast cancers that had undergone an epithelial-to-mesenchymal transition.

There was a statistically significant correlation between molecular subtype and sensitivity to PD 0332991 (χ^2 ^< 0.05). The subtypes most sensitive to growth inhibition by PD 0332991 were ER-positive. In addition, 10/16 HER2-amplified cell lines were sensitive. PD 0332991 inhibited growth in a cytostatic manner in these cells with no lethality observed (data not shown).

### Identification of differentially expressed genes and sensitivity to PD 0332991

**Figure 2:**
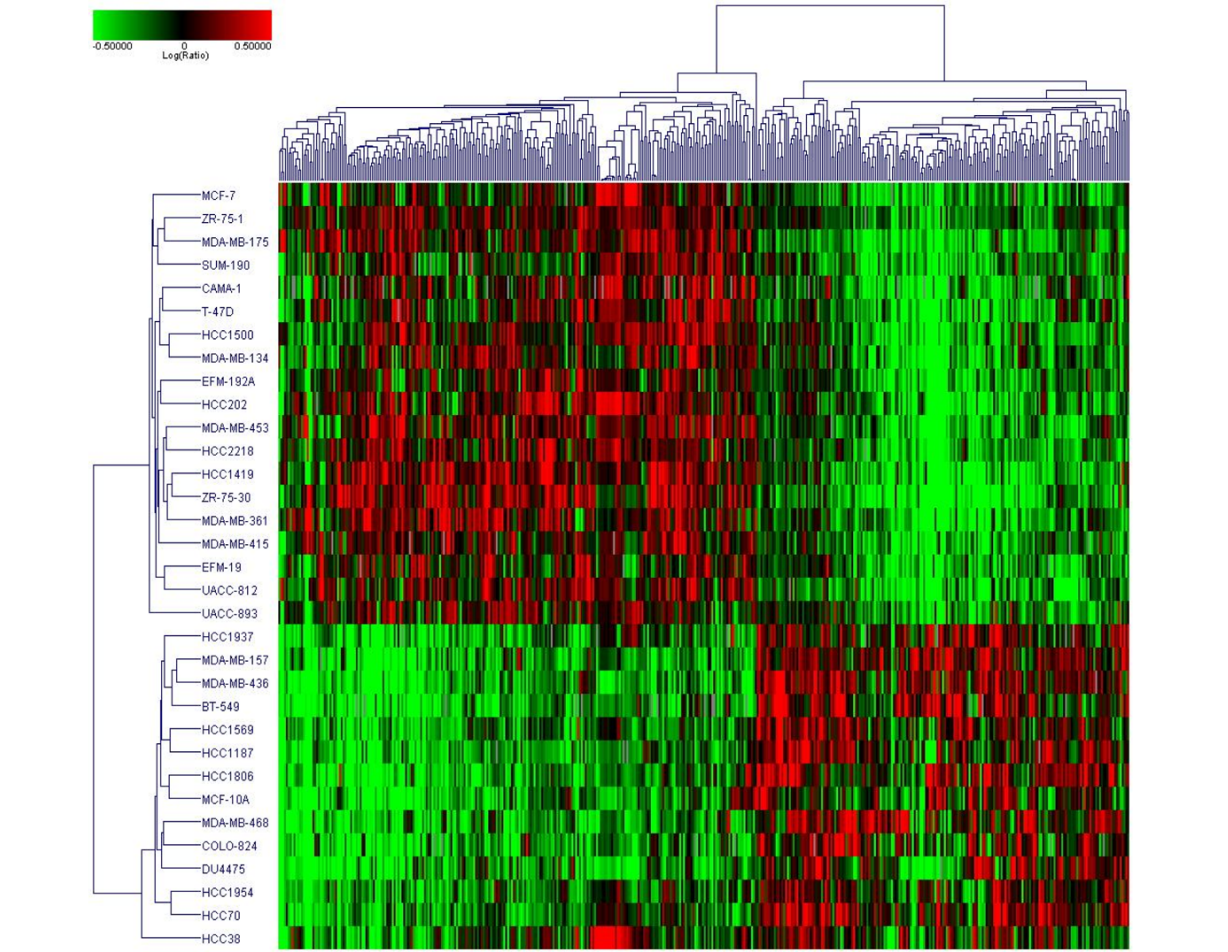
Differentially expressed genes between sensitive and resistant cell lines. Results of analysis of variance (ANOVA) identifying 450 differentially expressed genes between sensitive cell lines (IC_50 _< 150 nM) and resistant cell lines (IC_50 _> 1,000 nM). Retinoblastoma and cyclin D_1 _expression were higher in, and CDKN2A (p16) was lower in, sensitive cell lines. Full results of the ANOVA are available in the Additional data files.

Gene expression profiles were used to identify genes associated with response to PD 0332991. A set of 450 differentially expressed genes (*P *< 0.05, |sensitive group average - resistant group average| ≥ 0.2) was identified, where 253 genes were upregulated in sensitive cell lines and 197 genes were upregulated in resistant lines. Hierarchical clustering of the 43 cell lines across these 450 differentially expressed genes was performed (Figure 2).

A significant overlap was found between the gene set associated with response to PD 0332991 and the gene set that defines breast cancer cell subtypes. Of the genes more highly expressed in the sensitive cell lines, 193/253 (76%) are luminal markers and 0/253 are nonluminal markers. In the resistant gene set, 117/197 (59%) are nonluminal markers and 0/197 are luminal markers. RB1, cyclin D_1_, and CDKN2A (p16) were differentially expressed - with higher levels of RB1 and cyclin D_1_, and lower levels of p16, in the sensitive group. Of note, these three genes are cyclin related and are not in the breast cell type gene set.

Full results of the ANOVA with the exact genes and Venn diagrams demonstrating the overlap between the cell classification markers and response markers are available online (see Additional data files 1, 2, and 3). Importantly, both MCF-10A and HCC-38 - sensitive cell lines that are of nonluminal origin - cluster with the remaining sensitive lines when this operation is performed excluding the subtype marker set (see Additional data file 4), highlighting the robustness of the predictive marker set.

### PD 0332991 inhibits pRb phosphorylation in sensitive cell lines

It is known that CDK4/6 complexes with cyclin D_1 _to phosphorylate and inactivate pRb, thus allowing cell cycle progression. The effects of PD 0332991 on total pRb and Rb phosphorylation were determined using Western blot analysis of a subset of lines with variable sensitivities to PD 0332991.

**Figure 3:**
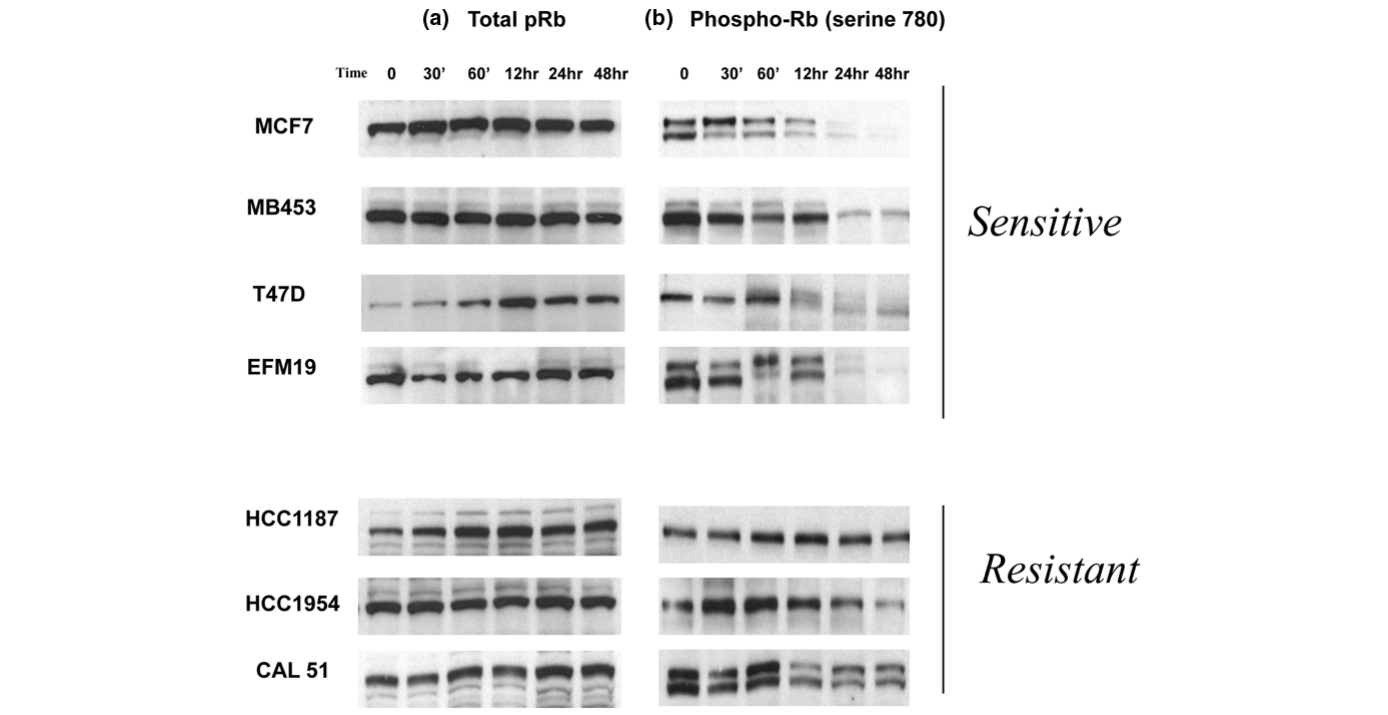
Effects of PD 0332991 on phosphorylation of retinoblastoma gene product. PD 0332991 blocks phosphorylation of retinoblastoma gene product pRb in sensitive cell lines but not in resistant cell lines. **(a) **In neither group does total pRb change significantly with treatment. **(b) **PD 0332991 significantly blocks phoshorylation of pRb (phospho-Rb) at serine 780 in sensitive cell lines (IC_50 _< 150 nM), but not in resistant cell lines (IC_50 _> 1,000 nM). All cell lines were treated with 100 nM PD 0332991 for the times specified and western blots were performed as described in Materials and methods.

There was no decrease in total pRB in either the sensitive group or the resistant group after treatment with the CDK4/6 inhibitor (Figure 3a). There were significant differences, however, in Rb phosphorylation when comparing sensitive and resistant cells after exposure to the compound. There was a rapid and sustained decrease in pRb with exposure to 100 nM PD 0332991 in the three more sensitive cell lines (Figure 3b). The majority of the less sensitive lines have lower amounts of total Rb, as determined by the microarray data; however, three resistant cell lines that have detectable Rb but are still resistant to PD 0332991 were also evaluated. These lines did not have a significant decrease in Rb phosphorylation with 100 nM PD 0332991.

### Effects of PD 0332991 on cell cycle and apoptosis

**Figure 4:**
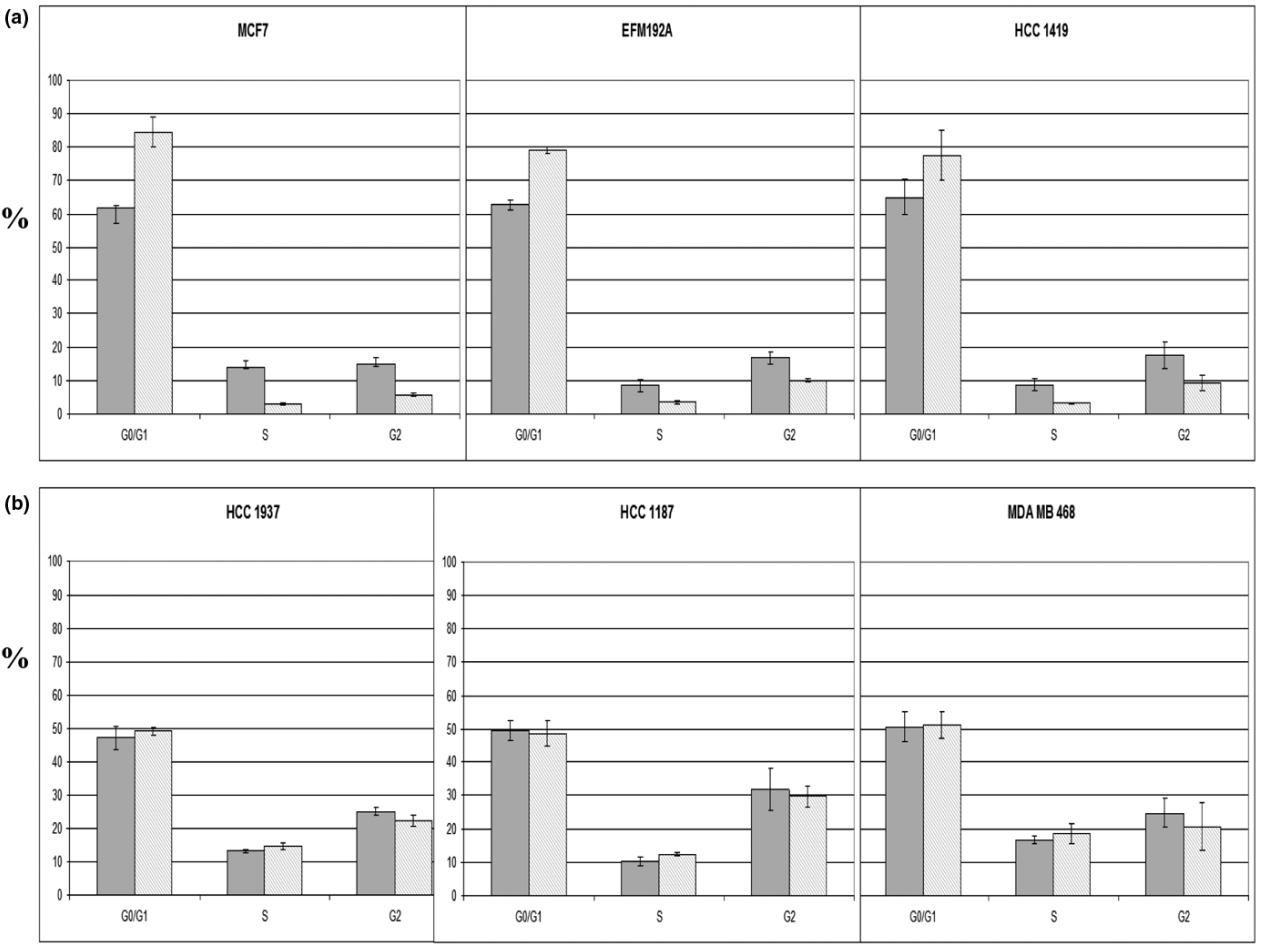
Effects of PD 0332991 on cell cycle. **(a) **Sensitive cell lines (IC_50 _< 150 nM) show marked G_0_/G_1 _arrest and a decrease in the S-phase fraction as compared with **(b) **resistant cell lines (IC_50 _> 1,000 nM) after incubation with 100 nM PD 0332991 for 24 hours. Solid bars, control samples; hatched bars, treated samples. Error bars represent the standard error for two separate experiments.

To evaluate the effects of PD 0332991 on the cell cycle and to correlate them with the antiproliferative effects of the compound, we treated a subset of both sensitive cell lines and resistant cell lines with PD 0332991 at 100 nM for 48 hours and then performed flow cytometry using Nim-DAPI staining. Clear and pronounced G_0_/G_1 _arrest was seen in cell lines that had lower IC_50 _values (IC_50 _< 150 nM) compared with those with higher IC_50 _values (IC_50 _> 1,000 nM) (Figure 4). There was no evidence of apoptosis in even the most sensitive cell lines when PD 0332991 was used as a single agent (data not shown).

Together, these data support a proposed cytostatic mechanism of action of this CDK4/6 inhibitor involving prevention of cell cycle progression by blocking hyperphosphorylation of pRb.

### Combinations of PD 0332991 plus tamoxifen and PD 0332991 plus trastuzumab in ER-positive and HER2-amplified breast cancer cells, respectively

**Figure 5:**
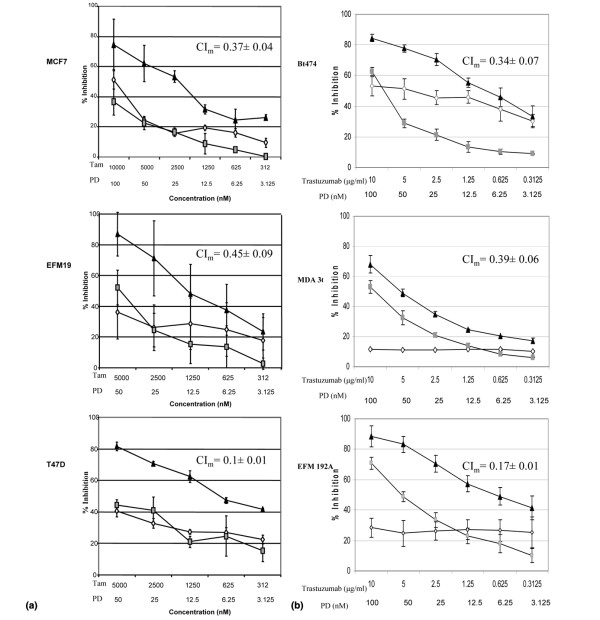
Effects of PD 0332991, tamoxifen, and trastuzumab on growth of breast cancer cell lines. PD 0332991 (PD) acts synergistically with tamoxifen (Tam) and trastuzumab in inhibiting growth of **(a) **estrogen receptor-positive human breast cancer cell lines and **(b) **HER2-amplified cell lines *in vitro*, respectively. (a) Grey squares, PD 0332991 alone; open diamonds, tamoxifen alone; dark triangles, PD 0332991 and tamoxifen combination. (b) Grey squares, PD 0332991 alone; open diamonds, trastuzumab alone; dark triangles, PD 0332991 and trastuzumab combination. Error bars represent the standard error for two separate experiments. Mean combination index (CI_m_) for the combination curves shown with standard error CI_m _< 1, indicating synergy for the combinations.

Given that both the ER-positive cell lines as well as HER2-amplified cell lines within the panel demonstrated greatest sensitivity to PD 0332991, the combination of targeted therapeutics plus the CDK4/6 inhibitor was evaluated in both of these molecular subtypes. In ER-positive breast cancer, hormonal blockade with tamoxifen is an effective treatment for early and advanced disease. For the three ER-positive lines evaluated, when considering the entire dose-response curve, the combination was synergistic with mean CI < 1 across clinically relevant concentrations of both drugs (Figure 5a). Effects of the combination on the cell cycle are shown in Additional data file 5.

Trastuzumab has been shown to have clinical activity in HER2-amplified breast cancer [34]. For the three HER2-amplified lines evaluated, again when considering the entire dose- response curve, the combination also proved to be synergistic with mean CI < 1 across clinically relevant concentrations of both drugs (Figure 5b). Effects of the combination on the cell cycle are shown in Additional data file 6.

### PD 0332991 overcomes acquired resistance to tamoxifen

**Figure 6:**
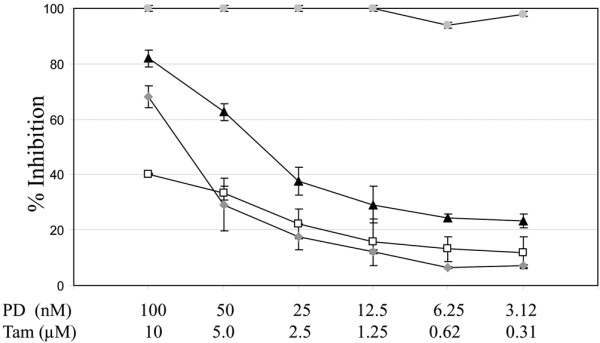
PD 0332991 and tamoxifen in a tamoxifen-insensitive cell line. PD 0332991 (PD) enhances the effects of tamoxifen (Tam) in an MCF7 tamoxifen-insensitive cell line. Dark triangles, PD 0332991 and tamoxifen combination; open squares, PD 0332991 alone; grey diamonds, tamoxifen alone; grey circles, MCF7 parental with tamoxifen alone. Error bars represent the standard error for two separate experiments. The MCF7 parental line is 100% inhibited under these conditions in the presence of tamoxifen (grey circles).

The MCF7 tamoxifen-resistant cell line has acquired tamoxifen resistance after serial passages in estrogen-free conditions. These MCF7 tamoxifen-resistant cells demonstrated sensitivity to monotherapy with PD 0332991. Treatment with PD 0332991 also enhanced sensitivity to tamoxifen in the resistant cells when the two agents were used in combination, although not restoring them to the level of the parental line (Figure 6).

## Discussion

The critical role of CDK-cyclin interactions in controlling cell growth has been an attractive target in cancer therapy for sometime. These data represent the most comprehensive preclinical evaluation of a CDK4/6 inhibitor in breast cancer cell lines to date, and build the case for its clinical development in specific molecular subgroups of breast cancer.

Using baseline Agilent gene expression profiles, we first demonstrated that luminal ER-positive and HER2-amplified breast cancer cell lines were more sensitive to CDK4/6 inhibition of proliferation and cell cycle arrest. ANOVA analysis of these data identified a set of genes that were associated with response to PD 0332991. While the majority of these genes were associated with the luminal subtype, increased RB1 and cyclin D_1 _as well as decreased CDKN2A (p16) were associated with sensitivity to the effects of PD 0332991 on the cell cycle and growth inhibition. Western blot analysis confirmed that Rb phosphorylation is decreased in sensitive cell lines after PD 0332991 treatment, while resistant lines that had detectable pRb were relatively resistant to the effects of PD 0332991 on Rb phosphorylation. In this case, the presence of pRb alone is not predictive of response to PD 0332991. pRb presence in the background of luminal ER-positive breast cancer, however, is predictive of response to the compound. pRb is present in some nonluminal breast cancer cell lines but these lines are resistant to both the antiproliferative effects of PD 0332991 and its ability to block Rb hyperphosphorylation.

Further studies will be required to determine why PD 0332991 cannot block hyperphosphorylation in cell lines that do contain pRb. One can speculate that potentially CDK4/6 is mutated in these cell lines and does not allow PD 0332991 binding and kinase inhibition, as is the case in resistance to BCR-ABL inhibitors in chronic myelogenous leukemia [35]. Alternatively, there may be another mechanism driving Rb hyperphosphorylation in resistant cell lines, such as a greater dependence on CDK1/2-cyclin E interactions or loss of negative regulators of this pathway in these cell lines.

Resistance to PD 0332991 in many of the nonluminal breast cancer cell lines may be explained by the absence of pRb. Recent publications highlighted the lack of pRb in basal-like breast cancer tissue [36] and observed that pRb depletion can result in the characteristic epithelial-to-mesenchymal transition changes seen in some breast cancer specimens [37], recapitulating our *in vitro *observations. The lack of activity of a CDK4/6 inhibitor in cell lines and tumors that lack pRb can be explained by the fact that cyclin D_1 _does not offer G_1 _control in the absence of pRb [38].

Published studies evaluating the role of cyclin D_1 _in breast cancer support the current observations of the activity of a CDK4/6 inhibitor in luminal ER-positive breast cancer, its synergism with tamoxifen in cell lines that are sensitive to hormone manipulation, as well as the reversal of resistance of those that have acquired a resistant phenotype in the face of anti-estrogen therapy. Estrogen effects on cell cycle progression are tightly linked to expression of cyclin D_1 _[39]. Cyclin D_1 _amplification and/or overexpression has been more commonly associated with an ER-positive breast cancer subtype [40] and, as mentioned previously, is associated with tamoxifen resistance [19,20]. Some studies suggest that overexpression of cyclin D_1 _can directly activate ER in a hormonally independent manner that is also independent of CDK and pRb function [21,22]. The data supporting this concept were reviewed recently [41]. In the large panel of human breast cancer lines we evaluated, however, 9/10 ER-positive lines were sensitive to PD 0332991 inhibition. Of interest, Wang and colleagues recently described a cyclin D_1 _splice variant - named cyclin D_1b _- that occurs in breast cancer tissue and cell lines, and whose expression can overcome cell cycle arrest induced by anti-estrogens via a CDK4 interaction [42].

In addition, a pivotal role for cyclin D_1 _function in HER2-mediated transformation has been described using transgenic mouse models [43]. More recently, the same authors defined that the ability of cyclin D_1 _to activate CDK4 is critical for driving tumorigenesis in these models. Moreover, CDK4-associated kinase activity is required to maintain breast tumorigenesis in this system [44,45] and a subset (~25%) of HER2-amplified breast cancers also have high cyclin D_1 _levels. The authors hypothesized that this 'subset may benefit from inhibiting CDK4 kinase' [44]. Our data would suggest that the benefit with PD 0332991 might be extended beyond that 25%, since the benefit was not dependent on elevated cyclin D_1 _alone in the HER2-amplified cell lines. In addition, synergistic efficacy was observed between trastuzumab and PD 0332991 that may also be independent of cyclin D_1 _measurement. Earlier work evaluating the nonspecific cyclin-CDK inhibitor flavopiridol in combination with trastuzumab also demonstrated similar activity in HER2-amplified cell lines [46]. Finally, a recent study investigating gene expression profiles of women with HER2-amplified breast cancer who develop early brain metastasis identified CDK4 expression as part of a 13-gene profile that predicted for early brain metastasis and death [47].

Acquired and *de novo *resistance to trastuzumab remains a management challenge in clinical oncology. While limited data exist about the role of CDK4-cyclin D_1 _interactions and trastuzumab resistance, these data suggest a role for dual targeting of the HER2 pathway and CDK4/6. Molecular profiling of the JIMT-1 human breast cancer cell line derived from a woman with progressive HER2-amplified disease while receiving trastuzumab did identify a small amplicon on 12q14.1, which contains the CDK4 gene [48].

## Conclusions

The goal of this preclinical study was to guide patient selection as this novel agent moves into the clinic. Given the data from the current study as well as published work, there is a strong rationale for clinical development of PD 0332991 focusing on ER-positive luminal breast cancer as well as HER2-amplified disease and combining CDK4/6 inhibition with anti-estrogen or anti-HER2 therapy, respectively.

## Abbreviations

ANOVA: analysis of variance; CDK: cyclin D kinase; CI: combination index; DMEM: Dulbecco's modified Eagle's medium; ER: estrogen receptor; FBS: fetal bovine serum; FITC: fluorescein isothiocyanate; IC_50_: concentration that inhibits growth by 50% of control; PBS: phosphate-buffered saline; PSF: 1% penicillin G-streptomycin-fungizone solution; Rb: retinoblastoma.

## Competing interests

RSF and DJS research funding from Pfizer, Inc. IC, CF, and GL are employees of Pfizer, Inc. The other authors declare that they have no competing interests.

## Authors' contributions

RSF and DJS designed and supervised the study, analyzed data, and drafted the manuscript. JD performed data analysis. DC, OK, DJC, and AD performed *in vitro *experiments and performed molecular biology. CG performed all microarrays and analyzed data. MA created the tamoxifen-resistant cell line and designed experiments. IC, CF, and GL collaborated in experimental design, data analysis, and manuscript writing. All authors read and approved the final manuscript.

## Supplementary Material

Additional file 1 Excel file containing a table that lists the genes from the ANOVA of sensitive cell lines versus resistant cell lines. Click here for file

Additional file 2 PowerPoint file containing a figure that shows a Venn diagram demonstrating the overlap between resistant and nonluminal markers in breast cancer cell lines. Click here for file

Additional file 3 PowerPoint file containing a figure that shows a Venn diagram demonstrating the overlap between sensitive and luminal markers in breast cancer cell lines. Click here for file

Additional file 4 PowerPoint file containing a figure that shows a cluster of differentially expressed genes between the sensitive and resistant cell lines that excludes genes associated with cell subtype. Click here for file

Additional file 5 PowerPoint file containing a figure that shows a cycle analysis of PD 0332991 in combination with tamoxifen. Click here for file

Additional file 6 PowerPoint file containing a figure that shows a cycle analysis of PD 0332991 in combination with trastuzumab. Click here for file
