# Supplementary material for: PD 0332991, a selective cyclin D kinase 4/6 inhibitor, preferentially inhibits proliferation of luminal estrogen receptor-positive human breast cancer cell lines in vitro
Source: Breast Cancer Res. 2009 Oct 29;11(5):R77. doi: 10.1186/bcr2419 (PMC2790859; doi:10.1186/bcr2419)
Supplement: Additional file 2 — PowerPoint file containing a figure that shows a Venn diagram demonstrating the overlap between resistant and nonluminal markers in breast cancer cell lines. [file bcr2419-S2.PPT]

## Slide 1
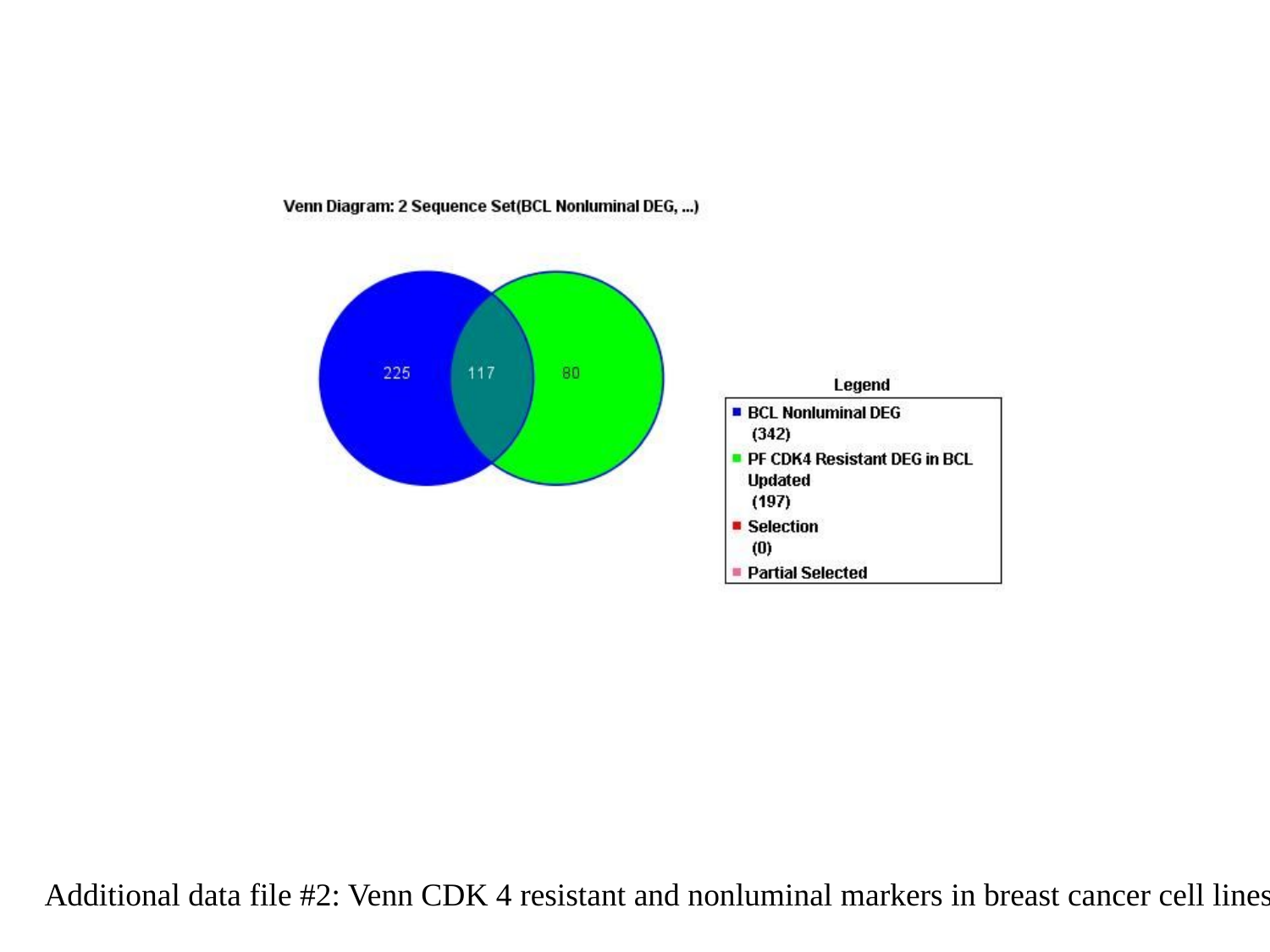

Additional data file #2: Venn CDK 4 resistant and nonluminal markers in breast cancer cell lines
