# Supplementary figures and images for: PD 0332991, a selective cyclin D kinase 4/6 inhibitor, preferentially inhibits proliferation of luminal estrogen receptor-positive human breast cancer cell lines in vitro
Source: Breast Cancer Res. 2009 Oct 29;11(5):R77. doi: 10.1186/bcr2419 (PMC2790859; doi:10.1186/bcr2419)

## Slide 1
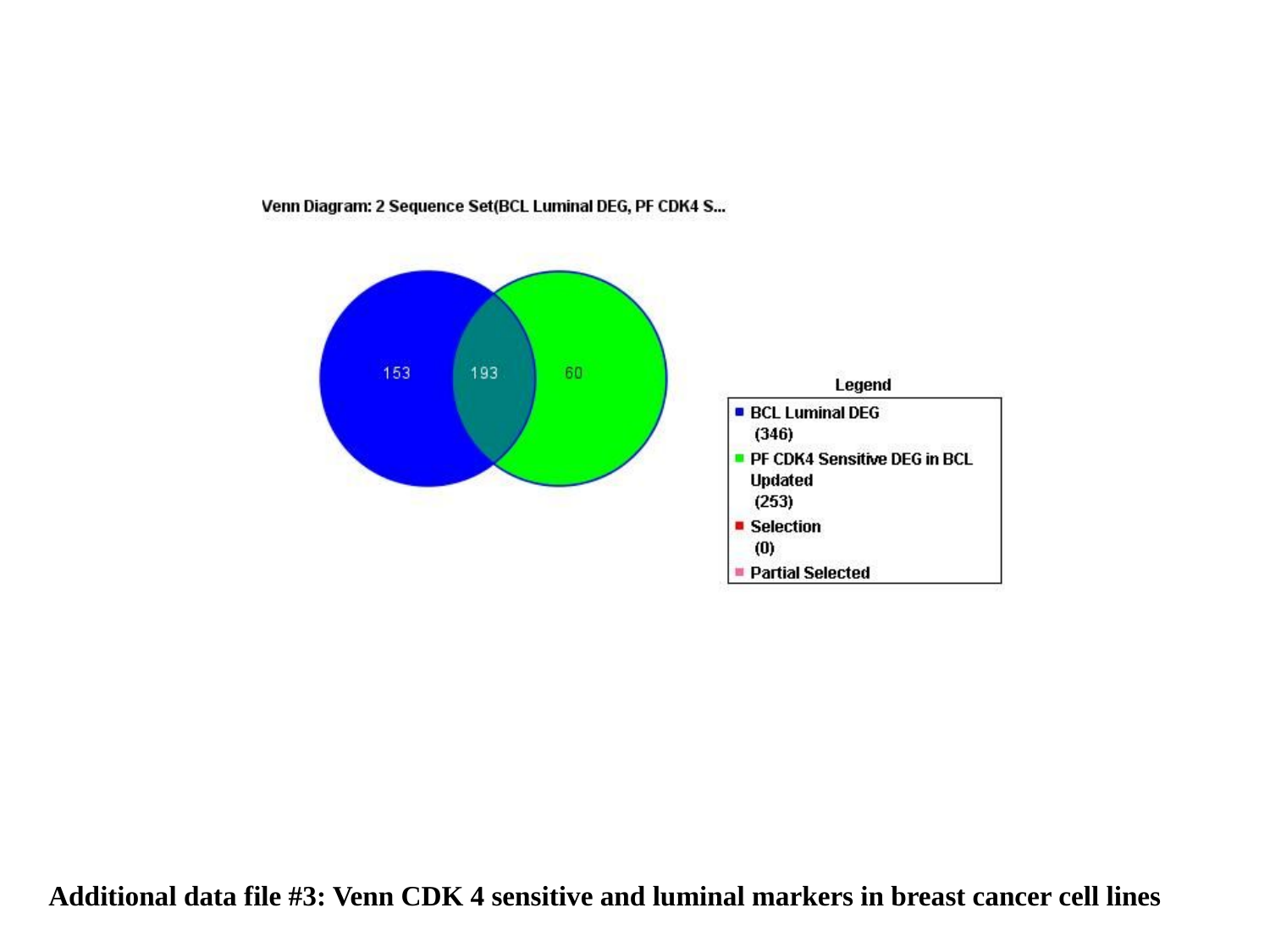

Additional data file #3: Venn CDK 4 sensitive and luminal markers in breast cancer cell lines

Supplement: Additional file 3 — PowerPoint file containing a figure that shows a Venn diagram demonstrating the overlap between sensitive and luminal markers in breast cancer cell lines. [file bcr2419-S3.PPT]

## Slide 1
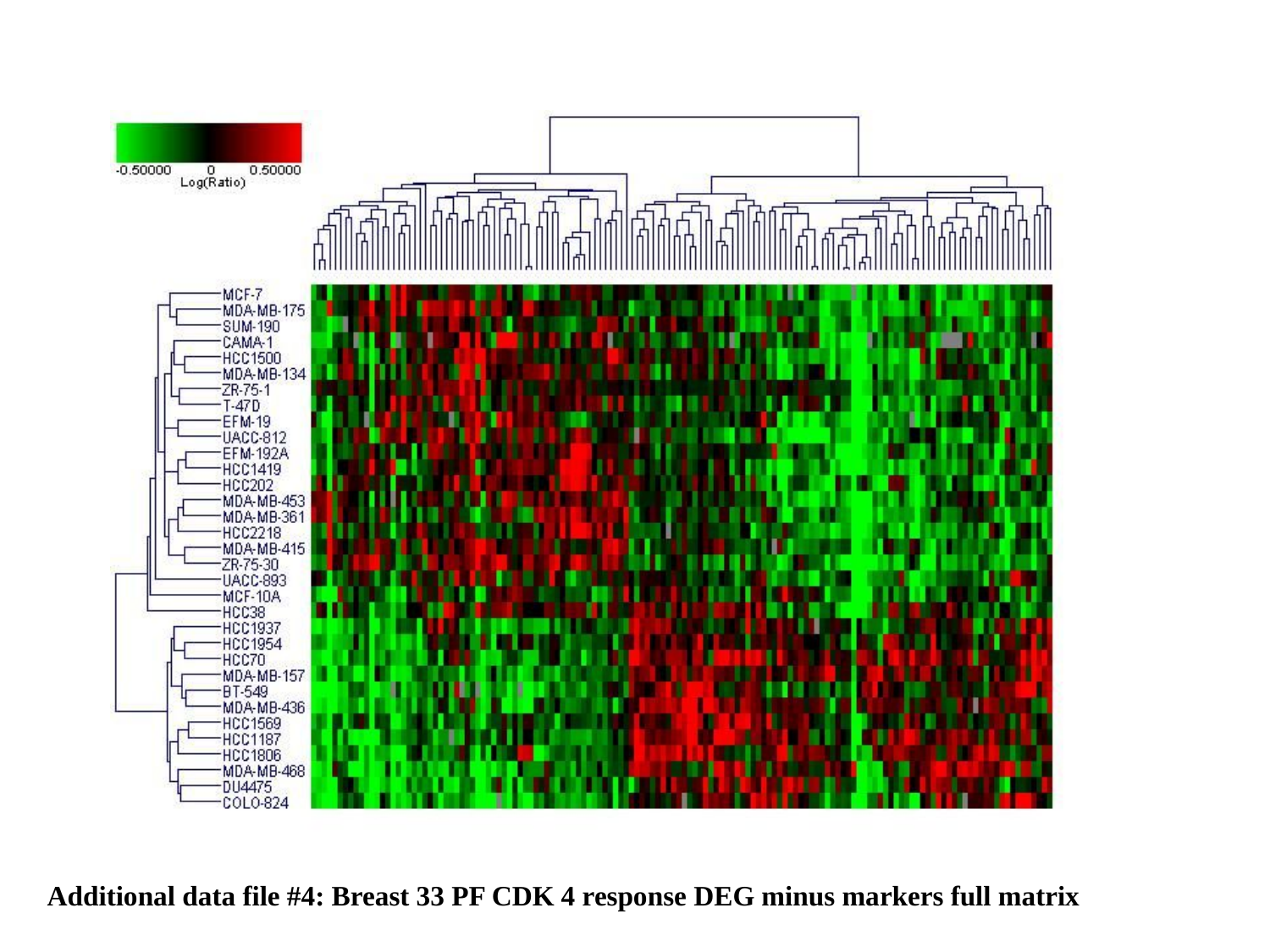

Additional data file #4: Breast 33 PF CDK 4 response DEG minus markers full matrix

Supplement: Additional file 4 — PowerPoint file containing a figure that shows a cluster of differentially expressed genes between the sensitive and resistant cell lines that excludes genes associated with cell subtype. [file bcr2419-S4.PPT]
