# Supplementary material for: PD 0332991, a selective cyclin D kinase 4/6 inhibitor, preferentially inhibits proliferation of luminal estrogen receptor-positive human breast cancer cell lines in vitro
Source: Breast Cancer Res. 2009 Oct 29;11(5):R77. doi: 10.1186/bcr2419 (PMC2790859; doi:10.1186/bcr2419)
Supplement: Additional file 5 — PowerPoint file containing a figure that shows a cycle analysis of PD 0332991 in combination with tamoxifen. [file bcr2419-S5.PPT]

## Slide 1
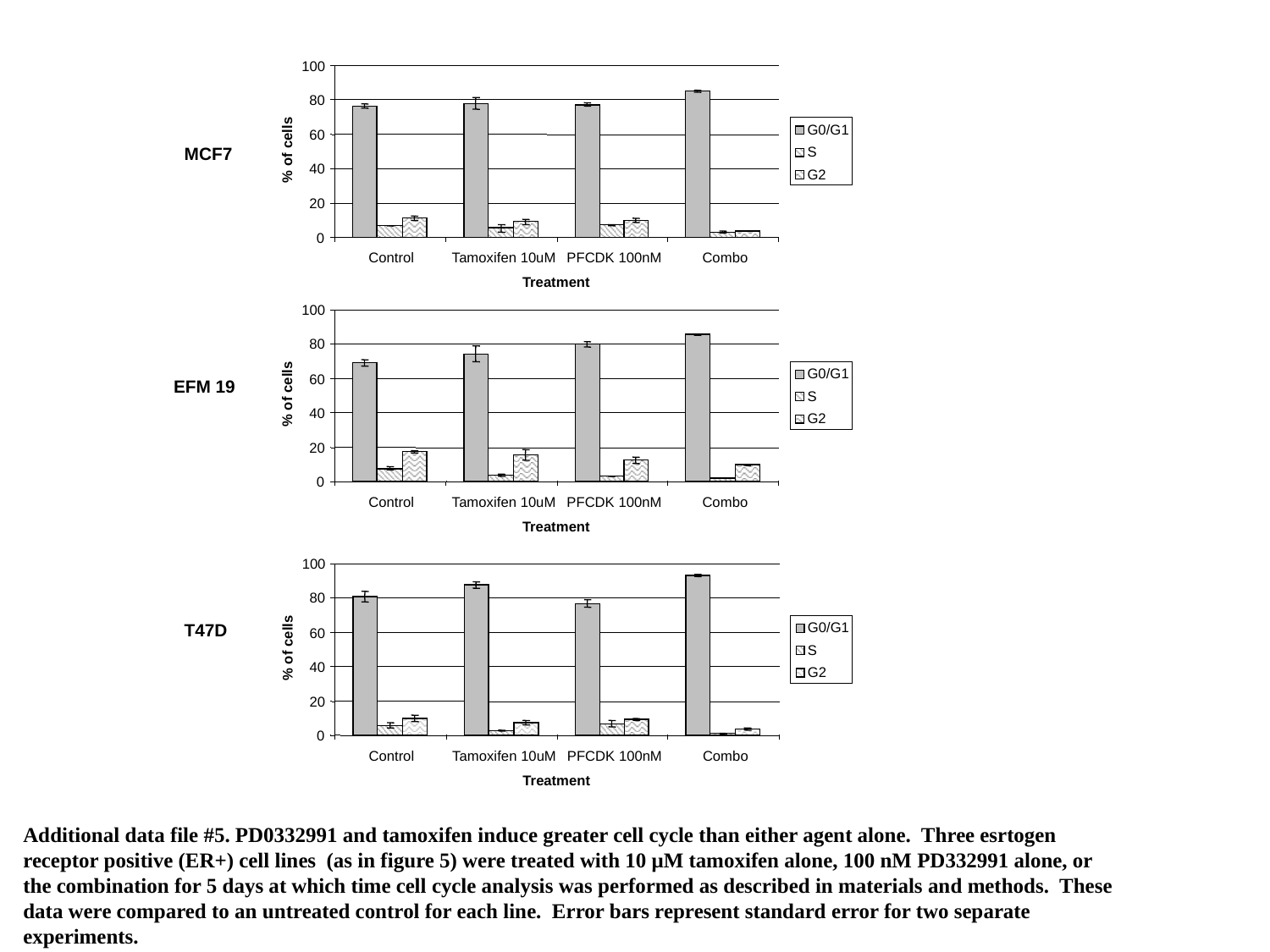

100
80
G0/G1
60
% of cells
MCF7
S
40
G2
20
0
Control
Tamoxifen 10uM
PFCDK 100nM
Combo
Treatment
100
80
G0/G1
60
EFM 19
% of cells
S
40
G2
20
0
Control
Tamoxifen 10uM
PFCDK 100nM
Combo
Treatment
100
80
G0/G1
T47D
60
% of cells
S
40
G2
20
0
Control
Tamoxifen 10uM
PFCDK 100nM
Combo
Treatment
Additional data file #5. PD0332991 and tamoxifen induce greater cell cycle than either agent alone. Three esrtogen receptor positive (ER+) cell lines (as in figure 5) were treated with 10 μM tamoxifen alone, 100 nM PD332991 alone, or the combination for 5 days at which time cell cycle analysis was performed as described in materials and methods. These data were compared to an untreated control for each line. Error bars represent standard error for two separate experiments.
