# Supplementary material for: PD 0332991, a selective cyclin D kinase 4/6 inhibitor, preferentially inhibits proliferation of luminal estrogen receptor-positive human breast cancer cell lines in vitro
Source: Breast Cancer Res. 2009 Oct 29;11(5):R77. doi: 10.1186/bcr2419 (PMC2790859; doi:10.1186/bcr2419)
Supplement: Additional file 6 — PowerPoint file containing a figure that shows a cycle analysis of PD 0332991 in combination with trastuzumab. [file bcr2419-S6.PPT]

## Slide 1
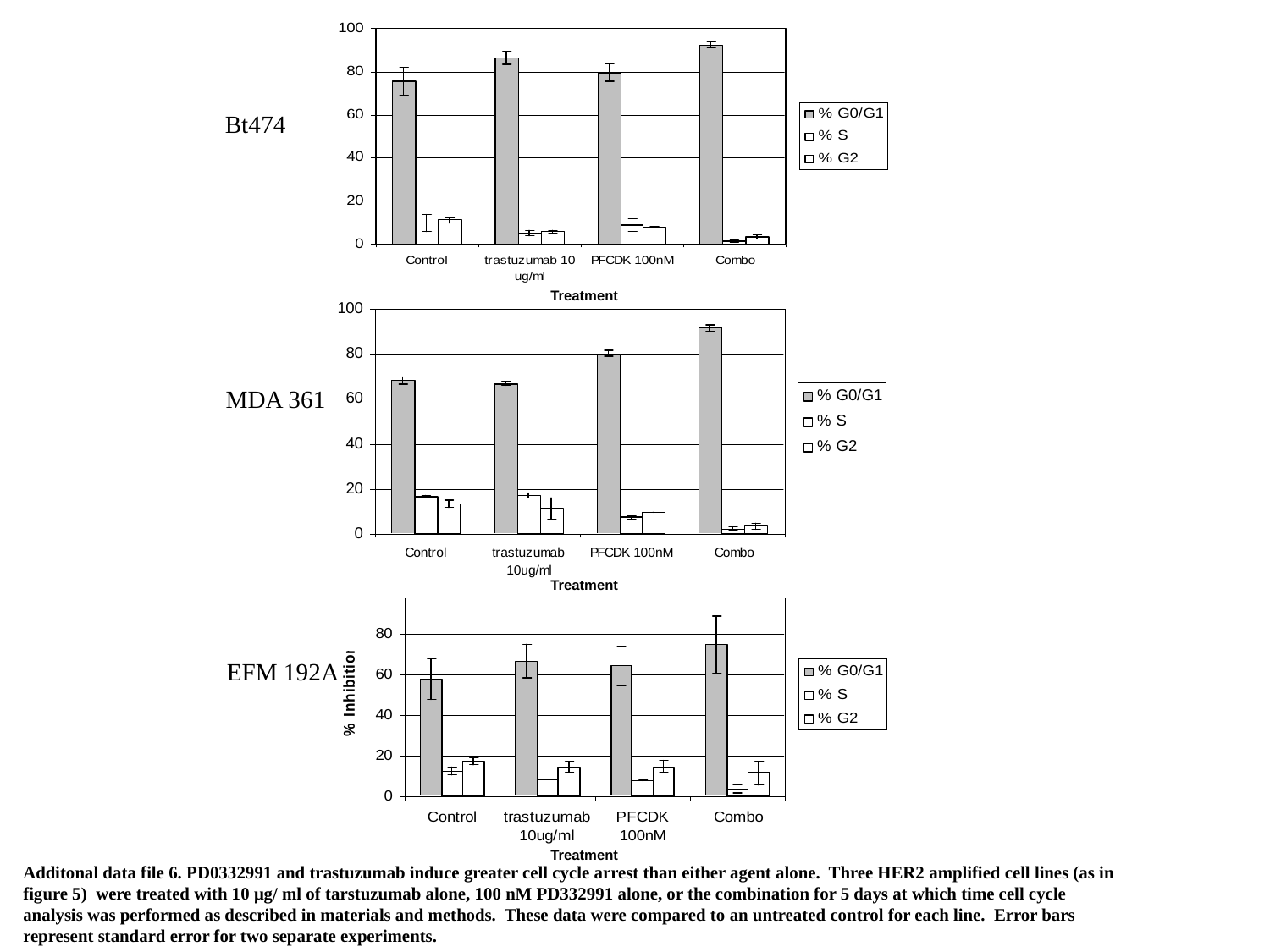

Bt474
Treatment
MDA 361
Treatment
EFM 192A
Treatment
Additonal data file 6. PD0332991 and trastuzumab induce greater cell cycle arrest than either agent alone. Three HER2 amplified cell lines (as in figure 5) were treated with 10 μg/ ml of tarstuzumab alone, 100 nM PD332991 alone, or the combination for 5 days at which time cell cycle analysis was performed as described in materials and methods. These data were compared to an untreated control for each line. Error bars represent standard error for two separate experiments.
